# Interaction of Heavy Metal Lead with Gut Microbiota: Implications for Autism Spectrum Disorder

**DOI:** 10.3390/biom13101549

**Published:** 2023-10-19

**Authors:** Yousef Tizabi, Samia Bennani, Nacer El Kouhen, Bruk Getachew, Michael Aschner

**Affiliations:** 1 Department of Pharmacology, Howard University College of Medicine, Washington, DC 20059, USA; 2 Faculty of Medicine and Pharmacy of Casablanca, Hassan II University, Casablanca 20100, Morocco; 3 Department of Molecular Pharmacology, Albert Einstein College of Medicine, Bronx, NY 10461, USA; michael.aschner@einsteinmed.edu

**Keywords:** lead toxicity, autism spectrum disorder, microbiota, gut–brain axis, probiotics

## Abstract

Autism Spectrum Disorder (ASD), a neurodevelopmental disorder characterized by persistent deficits in social interaction and communication, manifests in early childhood and is followed by restricted and stereotyped behaviors, interests, or activities in adolescence and adulthood (DSM-V). Although genetics and environmental factors have been implicated, the exact causes of ASD have yet to be fully characterized. New evidence suggests that dysbiosis or perturbation in gut microbiota (GM) and exposure to lead (Pb) may play important roles in ASD etiology. Pb is a toxic heavy metal that has been linked to a wide range of negative health outcomes, including anemia, encephalopathy, gastroenteric diseases, and, more importantly, cognitive and behavioral problems inherent to ASD. Pb exposure can disrupt GM, which is essential for maintaining overall health. GM, consisting of trillions of microorganisms, has been shown to play a crucial role in the development of various physiological and psychological functions. GM interacts with the brain in a bidirectional manner referred to as the “Gut–Brain Axis (GBA)”. In this review, following a general overview of ASD and GM, the interaction of Pb with GM in the context of ASD is emphasized. The potential exploitation of this interaction for therapeutic purposes is also touched upon.

## 1. Introduction

Autism Spectrum Disorder (ASD) is a neurodevelopmental disorder characterized by persistent deficits in social interaction and social communication, as well as restricted and repetitive patterns of behavior that manifest in early childhood (DSM-V). This is followed by significant impairment in social and occupational functions in adolescence and adulthood that continues throughout the lifespan. Although the exact causes of ASD have yet to be fully characterized, there is growing evidence that multiple factors, including genetic factors, gut microbiota (GM), and environmental exposure to toxic substances such as lead (Pb), play important roles in its etiology. Pb is a toxic heavy metal that has been linked to a wide range of negative health outcomes, including anemia, encephalopathy, gastroenteric diseases, and, more importantly, cognitive and behavioral problems. Recent studies have revealed that Pb exposure can disrupt GM, which is essential for maintaining overall health. GM, which consists of trillions of microorganisms, has been shown to play a crucial role in the development of various physiological and psychological functions. Indeed, its disruption, commonly referred to as dysbiosis, has been linked to numerous neurologic diseases, including ASD. This review provides an overview of the current understanding of the relationship between Pb, GM, and ASD. It also identifies the potential exploitation of this knowledge for novel therapeutic interventions in ASD.

## 2. Autism Spectrum Disorder (ASD)

ASD is a major medical and social challenge not only for those diagnosed but also for their caregivers and families. It is a developmental disability affecting social interaction and communication, as well as sensory processing and behavior. The severity of ASD is mainly based on the level of social interaction, communication, and behavioral impairments described in detail below. Although intellectual disability is not a diagnostic criterion, when present, however, it imparts an added challenge for the caregivers and the patient. Genetically, it is highly heterogeneous and may be either inherited or caused by novel mutations. To date, hundreds of genes have been identified that may contribute to the serious deficits in communication, social cognition, and behavior associated with ASD. However, these identified genes may account for 10–20% of all ASD cases. Thus, a combination of genetics, epigenetics, environmental factors, and the immune system are considered central players in ASD susceptibility [1,2]. Moreover, significant variations in symptoms, despite similar pathogenic changes, may be observed [1]. According to the American Psychiatric Association’s *Diagnostic and Statistical Manual* (DSM-V), for a child to meet diagnostic criteria for ASD, they must have persistent deficits in each of three areas of social communication and interaction (deficits in social-emotional reciprocity; deficits in non-verbal communicative behaviors; and deficits in developing, maintaining, and understanding relationships), plus at least two of four types of restricted, repetitive behaviors (stereotyped motor movements and use of speech; inflexible adherence to routines and patterns; fixated interests with abnormal intensity and focus; or hyperactivity and hypo- or hyper-reactivity to sensory input). Indeed, most individuals diagnosed with ASD have unique and atypical sensory experiences, especially regarding tactile, visual, and auditory stimuli [3]. Curiously, ASD shares some of the symptoms consistent with attention-deficit/hyperactivity disorder (ADHD), namely inattention, hyperactivity, and impulsivity [4,5].

Due to improved access to healthcare facilities and due to the growing awareness of mental health, the prevalence of ASD has increased in recent years and has become a cause of major concern [6]. ASD is about three times more prevalent in boys than girls. In the United States (US), about 1 in every 42 males and 1 in 189 females is diagnosed with ASD [7]. Most children are diagnosed after age 4, although detection may be feasible as early as age 2. Early detection and intervention are crucial for providing improvement opportunities. Behavioral, speech, language, occupational, and social skills training are the main approaches applied. The effectiveness of early intervention with positive outcomes, especially in the realm of cognitive ability; daily living skills and motor skills were recently reported as well [8]. These individuals also have an increased risk of other psychiatric conditions, such as anxiety and eating disorders. Unfortunately, the spectrum of neurodevelopmental disabilities persists throughout life, and as of yet, beyond modest symptomatic relief, no cure is available. Treatment strategies often include behavioral, physical, occupational, and speech therapy.

### 2.1. Neurobiological Substrates Associated with ASD

The neurobiological substrates associated with ASD are not fully understood, but several studies have identified brain regions and neural pathways that might be involved in its pathophysiology. One of the most consistent findings is that individuals with ASD present with structural and functional differences compared to individuals without ASD. These include smaller cell size and increased density of cells in the hippocampus, limbic system, entorhinal cortex, and amygdala, abnormal development of frontal and temporal lobes, as well as lower gray matter and white matter in all ages of individuals suffering from ASD. However, younger patients tend to manifest an increase in neuronal size in the cerebellar nuclei, inferior olive, and vertical limb of Broca’s area [9,10]. More recently, structural Magnetic Resonance Imaging (MRI) studies dealing with total brain volume, regional brain structure, cortical area, and task-based functional MRI (fMRI) show dysfunctional activation in critical areas controlling social communication and restrictive repetitive behavior [11]. The current hypothesis suggests that ASD symptomatology is driven by four brain regions controlling social behavior. These include amygdala, orbitofrontal cortex (OFC), temporoparietal cortex (TPC), and insula [12]. Specifically, the amygdala’s contribution to ASD is due to its major involvement in intangible knowledge representations and gaze guidance. In addition, the disruption of interconnected circuitries in the visual cortex, inferior frontal gyrus, caudate nucleus, and hippocampus can contribute to further cognitive and other symptoms [12]. A recent study reported that autistic individuals showed different atypical regional gray matter volumetric changes in childhood, adolescence, and adulthood compared to their control peers, indicating that it is essential to consider developmental stages of the brain when exploring brain structural abnormalities in autism [13].

### 2.2. Genetics and Epigenetics in ASD

As mentioned above, genetics and epigenetics also play an important role in the development of ASD, and several genes, including those involved in synaptic function, neuronal signaling, and brain development, have been implicated. Some of the other genes in which rare mutations are associated with ASD, often with other signs and symptoms, are ARID1B, ASH1L, CHD2, CHD8, DYRK1A, POGZ, SHANK3, and SYNGAP1. In most individuals where ASD is caused by rare gene mutations, the mutations occur in only a single gene. Single nucleotide polymorphisms (SNPs) of a number of candidate genes, including CNTNAP2, MTHFR, OXTR, SLC25A12, and VDR, have been identified [14,15]. Some of the candidate genes responsible for 90–95% of all idiopathic autism cases and related to brain metabolism include AVPR1a, DISC1, DYX1C1, ITGB3, SLC6A4, RELN, RPL10, and SHANK3 [15].

Regarding epigenetics, hundreds of potential environmental factors that may contribute to ASD risk have been identified [1]. These include advanced maternal and paternal age, maternal complications or infections during pregnancy, prenatal exposure to anticonvulsants, particularly valproic acid, toxic chemical exposure, smoking and alcohol use, nutrition, maternal diabetes, enhanced steroidogenic activity, immune activation, altered zinc–copper cycles and treatment with selective serotonin reuptake inhibitors [16]. It is noteworthy that epidemiological studies demonstrate no evidence of vaccination posing an autism risk [16]. On the other hand, twin studies involving 50 pairs of monozygotic twins discordant for ASD confirm autism-associated differentially methylated regions, with methylation patterns at some CpG sites common to symptom groups [17]. It is relevant to mention that there are four different ways epigenetics can promote or inhibit gene expression. These consist of histone modifications or deacetylation, DNA methylation, RNA interference, and RNA modifications [18]. Among others, increased DNA methylation of the GAD1 gene promoter was described in patients with ASD as an example of the role of epigenetics in ASD pathophysiology [19].

In short, it can be concluded that ASD is a multifactorial disorder in which genetic and environmental factors interact, triggering its development. However, precise causal mechanisms remain to be elucidated.

### 2.3. Neurotransmitters in ASD

Given the complexity of ASD and structural brain abnormalities, it is not surprising that a plethora of neurotransmitters, including GABA, glutamate, serotonin, dopamine, acetylcholine, and several peptides such as oxytocin, arginine-vasopressin, melatonin, orexin, opioids, as well as N-acetyl aspartate and vitamin D have been implicated in its etiology [20]. Moreover, neurotransmitter deficiencies in ASD patients were recently verified by functional magnetic resonance imaging [21]. A recent review covers the specific involvement of each of the above transmitters in detail [20]. Here, we provide a brief discussion of some of the more important players with an emphasis on therapeutic potentials.

Glutamate, the main excitatory neurotransmitter in the brain, targets both metabotropic (mGluR) and ionotropic (iGluR) receptors. The metabotropic receptors are G-protein coupled receptors (GPCR), whereas ionotropic receptors are ligand-gated ion channels. To date, eight different types of mGluRs have been identified. The three ionotropic receptors consist of N-methyl-D-aspartate receptors (NMDARs), α-amino-3-hydroxy-5-methyl-4-isoxazolepropionic acid receptors (AMPARs), and kainate receptors. Both mGluRs and iGluRs play an important role in synaptic plasticity, which is important for learning and memory. Of mGluRs, mGluR1 and mGluR5 are the most studied in ASD. Curiously, altered functioning of the mGluR5 receptor also occurs in Fragile X syndrome, which is a major genetic cause of autism and is associated with the most common ASD phenotypes. Genetic alterations have also been linked to the NMDA class of iGluRs, where both NMDA hyperfunction and hypofunction are associated with an ASD phenotype. Indeed, valproic acid, which causes an overexpression of NMDA receptors, has been used to model autism in rodents. Reduced AMPA receptors have also been observed in ASD. Since the onset of ASD symptoms coincides with the timing of synapse formation and maturation, and glutamatergic receptors are key players in this scenario, a causative association between GluRs and ASD is suggested [22].

Gamma-aminobutyric acid (GABA), derived from glutamate, is the major inhibitory neurotransmitter in the mature brain and has a complex balancing act with its precursor. GABA also acts on both traditional ionotropic and metabotropic receptors. However, the functional properties of GABA receptor signaling in the immature brain are significantly different from, and in some ways opposite to those found in the adult brain (i.e., major inhibitory neurotransmitter). Thus, in the immature brain, GABA may act as the main excitatory neurotransmitter or a trophic factor influencing proliferation, migration, differentiation, synapse maturation, and cell death [23]. This unique feature of the early GABA signaling, as well as the excitatory/inhibitory imbalance theory concerning abnormal social behavior [24], signify GABA’s important role as a developmental signal and a major player not only in ASD etiology but also in symptom manifestations such as impairment in information processing and social behavior dysfunction [20]. This contention was supported in a recent preclinical study highlighting an impaired GABA release, as well as a decrease in both GABA A and GABA B receptor subunit expression in the valproic acid model of ASD [25].

Serotonin (5-hydroxytryptamine, 5-HT) is an important neurotransmitter involved in several developmental events, including cell division, cortical proliferation, migration, differentiation, cortical plasticity, and synaptogenesis. Its role in various brain functions such as mood regulation, learning and memory, and sleep is well established. Indeed, selective serotonin reuptake inhibitors are the most used antidepressants. In relation to ASD, higher levels of 5HT have been detected in autistic children and in animal models of ASD, while reductions in both 5-HT2A and 5-HT1A binding were observed in postmortem ASD brains [26,27]. The involvement of the 5HT system in the etiology of ASD during early brain development has been suggested [28].

Dopamine (DA), a major catecholamine neurotransmitter, in addition to its well-established roles in motor control and neuronal regulation of prolactin, is critically involved in reward circuitry and addictive behaviors, as well as in social cognition and behavior. Its link to ASD has been suggested by numerous studies. In fact, it has been hypothesized that dopamine imbalances in specific brain regions could lead to ASD [29]. Specifically, reduced dopamine release in the nucleus accumbens and prefrontal cortex has been reported in autistic subjects. These and other findings have led to the suggestion that social deficits in ASD are due to a dysfunction of the mesocorticolimbic dopaminergic circuit, while the dysfunction of the nigrostriatal dopaminergic circuit is responsible for the stereotype behaviors. Interestingly, administration of D1 dopaminergic receptor antagonists is effective in reducing stereotype behavior [20]. More recent imaging studies have confirmed monoamine neurotransmitter deficits in the cerebrospinal fluid of ASD patients [21].

Acetylcholine (ACh) is another excitatory neurotransmitter with extensive peripheral, as well as central functions. Thus, it is used by motor neurons at the neuromuscular junction and acts at both sympathetic and parasympathetic preganglionic neurons in the autonomic nervous system. It serves as the neurotransmitter at all the parasympathetic innervated organs and as the neurotransmitter at several sympathetic-innervated organs such as the sweat glands and the piloerector muscle. Importantly, it is considered a major neurotransmitter in the brain with an essential role in cognitive, motor, and other important behavioral functions such as social interactions [30]. ACh actions are mediated by both muscarinic, which are GPCRs, and nicotinic receptors, which are ligand-gated ion channels, whereas at the neuromuscular junction and the autonomic ganglia, nicotinic receptors are the sole mediators of the ACh. In the central nervous system (CNS), both nicotinic and muscarinic receptors are at play. The more abundant nicotinic receptors in CNS consist of α4β2 and homomeric α7 nAChRs. The main evidence of cholinergic system abnormalities in ASD includes a significant reduction in the nicotinic α4β2 subtype in the parietal and frontal, as well as a reduction in cerebellar α4 nAChRs, which could be linked to the loss of Purkinje cells and to a compensatory increase in α7 nAChRs [20,31]. Moreover, the administration of ABT-418, a neuronal nicotinic acetylcholine receptor agonist, imparts a statistically significant improvement in ASD-associated psychiatric symptoms [32].

The α7 nicotinic receptor also has a promising role in the pathogenesis of ASD and other related neuropsychiatric disorders such as ADHD, as it is involved in sensory processing, cognition, working memory, and attention and is highly expressed in the hippocampus and frontal cortex, regions involved in cognitive functions [33,34]. In addition, the therapeutic potential of α7 nicotinic acetylcholine receptor agonists in ASD or Down syndrome has been suggested [35].

**Figure 1:**
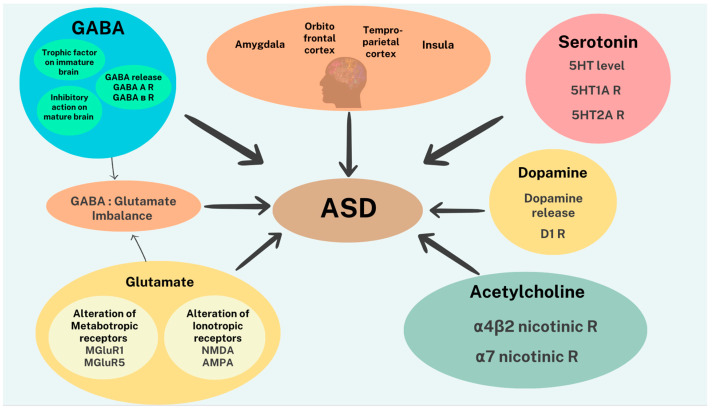
Schematic diagram depicting neurobiological substrates of ASD in relation to the brain areas and neurotransmitters and receptors implicated.

In summary, it may be suggested that dysfunction of any or a combination of the above-discussed neurotransmitters may contribute to both the etiology and/or symptomatology of ASD (Figure 1).

### 2.4. Neuroinflammation in ASD

Several meta-analyses have confirmed a strong involvement of neuroinflammatory processes in practically all neuropsychiatric and/or neurodegenerative diseases, including ASD [2,36,37,38,39,40,41,42,43,44,45]. Thus, it has been reported that individuals with ASD have significantly higher levels of several inflammatory biomarkers, including tumor necrosis factor-alpha (TNF-α), interleukin-1 beta (IL-1β), and C-reactive protein (CRP) compared to healthy controls [38]. Although the exact triggers and/or players in neuroinflammation associated with specific neurodegenerative/neuropsychiatric diseases have yet to be fully characterized, a central role for microglia and the commonality of immune dysfunction is well-recognized [46]. There is evidence of innate immune dysfunction, including aberrant innate cellular function leading to microglial activation and neuroinflammation [42].

As mentioned earlier, no cure for ASD is yet available. However, research in this area and the potential use of anti-inflammatory drugs and antioxidants, as well as mesenchymal stem cell-based therapy, have been suggested [41,45]. Recently, the involvement of the histaminergic system and potential therapeutic exploitation of this system in ASD and other conditions associated with impairment in executive functioning was provided [40]. Therefore, from the above discussions, it may be deduced that an imbalance in neurotransmitter systems and/or induction of neuroinflammation could precipitate ASD.

Moreover, a role for the gut–brain axis involving microbial–immune–neuronal crosstalk leading to neuroinflammation has been established [39]. Below, further contribution of this axis and its potential therapeutic exploitation is elaborated.

## 3. Gut Microbiota

The human gastrointestinal (GI) tract harbors a complex and dynamic population of microorganisms. The collection of bacteria, archaea, and eukarya colonizing the GI tract is termed the “gut microbiota”, which has co-evolved with the host over thousands of years to form an intricate and symbiotic relationship [47]. It has been estimated that the human GI tract harbors a range of 2000 bacterial species and was thought to contain over 100 times the amount of genomic content (microbiome) compared to the human genome [48,49]. However, more recent studies suggest only a slightly higher number of microbiomes compared to the human genome [50,51,52]. The gut microbiome is now commonly referred to as a new metabolic “organ” due to its immense impact on human equilibrium, including host metabolism (e.g., by converting inaccessible nutrient sources, such as plant polysaccharides and other complex carbohydrates, into readily absorbable metabolites) [53,54], physiology, nutrition, immune function (e.g., by inducing and training the host immune system [55]. In return, the immune system has largely evolved to maintain the symbiotic relationship of the host with these highly diverse and evolving microbes. Neurology, mental health, and aging process [52,55,56,57].

Vitamin B6 (VB6) compounds include pyridoxine and its derivative, pyridoxine-5-phosphate; pyridoxamine and its derivative, pyridoxamine-5-phosphate (P5P); pyridoxal and its derivative, pyridoxal-5-phosphate (PLP). PLP, which is used to assess VB6 in the blood, acts as a coenzyme in numerous reactions, including transamination and decarboxylation. Deficiency in vitamin B6 can lead to anemia, peripheral convulsions, neuropathy, hyper-irritability, and pellagra-like syndrome. Prolonged deficiency can lead to confusion, depression, and seizures, as well as abnormalities in EEG. A deficiency of P5P was detected in the urine of children with ASD, further supporting a role for VB6 and dysbiosis in ASD [58]. Indeed, it was recently reported that VB6 deficiency in rats induces ASD-like behaviors, such as impairment in social interactions and increased bowel frequency. It was further postulated that this might be due to dysregulated autophagy in the hippocampus [59]. VB6 is also considered a methyl nutrient that plays the role of both substrate and cofactor in transformations related to one-carbon metabolism. VB6 deficiency can lead to disruption in s-adenosyl-methionine (SAM) synthesis, which is a primary donor of the methyl group in the DNA methylation process [60]. DNA methylation, a reversible epigenetic modification that plays a crucial role in transcriptional gene silencing, can be harmful if excessive (hypermethylation) or insufficient (hypomethylation). However, the effect of supplementation of methyl nutrients on the DNA methylation process is inconclusive [60]. Nonetheless, Chen et al. (2023) provide justification for the use of VB6 supplementation for the treatment of ASD [59].

### 3.1. Digestive System Innervation—Enteric Nervous System

The digestive system is innervated by the CNS and by the enteric nervous system (ENS) that is within the wall of the gastrointestinal tract. The ENS plays a crucial role in controlling many of the functions of the digestive system, including the movement of food through the intestines, the secretion of digestive enzymes and hormones, and the sensing of nutrients and other signals in the gut. In fact, many neurons are contained in the enteric nervous system, about 200–600 million in a human. This is more than the total number of neurons of all sympathetic and parasympathetic ganglia combined and about the same number of neurons that are in the spinal cord. The intestinal microbiota plays an important role in regulating gastrointestinal structure and function via interactions with ENS. Indeed, GM helps enteric neuronal survival via lipopolysaccharide (LPS) and promotes neurogenesis via short-chain fatty acids (SCFAs). For this reason, it has been suggested that therapeutic developments for the treatment of enteric neuropathies may be achieved via GM manipulation [61].

The mechanism by which the ENS controls digestive function is complex and involves many different types of neurons and signaling molecules. ENS is composed of thousands of small ganglia that lie within the walls of the esophagus, stomach, small and large intestines, pancreas, gallbladder, and biliary tree. Two major sets of ganglia are found: the myenteric (Auerbach) plexus between the external muscle layers and the submucosal plexus (Meissner) that regulates the gut function. The neurons of the myenteric plexus are responsible for regulating the contractions of the digestive muscles, controlling the speed and force of peristalsis, and ensuring the efficient mixing and propulsion of food through the digestive tract. These neurons use a variety of neurotransmitters, including acetylcholine, serotonin, dopamine, and nitric oxide, to communicate with each other and with other parts of the digestive system. The submucosal plexus, on the other hand, is present in the small and large intestines. Its neurons are responsible for regulating the secretion of digestive juices and enzymes, controlling the blood flow to the digestive organs, and transmitting signals to the myenteric plexus and the CNS about the state of the digestive system. Most recently, it was revealed that enteric glia, located along nerve fibers in the gut mucosa, influences several important features of the gut epithelium, including barrier integrity, ion transport, and capacity for self-renewal [62]. Moreover, enteric glia also interacts with the endocrine and immune cells within the intestinal wall to maintain general homeostasis [63].

### 3.2. Gut–Brain Axis (GBA)

ENS is sometimes referred to as the “second brain” because it can operate independently of CNS and can coordinate complex digestive functions even in the absence of CNS input. However, the ENS also receives input from the CNS and can communicate bidirectionally with the brain via the vagus nerve. Additionally, the brain and the gut can communicate via the endocrine and immune systems involving the gut hormones and cytokines, which are under the direct influence of GM. Microbiota is also essential for maintaining the ENS integrity [61]. Thus, the relatively new concept commonly referred to as the “Gut–Brain Axis (GBA)” encompasses the bidirectional communication between the brain and the gut, primarily involving GM.

Recent findings suggest that GBA represents a complex interplay that is crucial in CNS development both pre- and post-natally. This hypothesis is supported by a series of experiments in germ-free mouse models where development in specific parts of CNS, crucial in cognitive and emotional responses, such as hippocampus and amygdala, is compromised in these mice [64,65]. Moreover, germ-free mice have an abnormal response to stress, which can be normalized by recolonizing them with a complete microbiota (via stool transplant) or by monocolonization with *Bifidobacterium Infantis* [66]. It is now well recognized that GM can affect the integrity and the diverse functions of CNS, including regulation of mood and cognition [67,68,69,70].

### 3.3. Gut Microbiota—Genetics

In addition to the well-known effects of environmental factors on the composition of the microbiota, host genetics also influences this composition [71]. It has been shown that monozygotic twins have more similar GM compared to dizygotic twins, strengthening the role of genes in GM [72]. Nonetheless, environmental factors such as diet, antibiotics, and stress affect the microbiome more profoundly than the genetic component. It is estimated that human genetics could explain approximately 2–8% of gut microbiome variation [71,73,74], whereas colonization of the gut by microbes, especially in early life, is largely determined by environmental factors such as diet and even delivery mode (e.g., vaginal vs. cesarean birth) [75].

Considerable effort is currently expended on identifying specific genes involved in both ASD and microbiota. It was reported recently that children who had certain genetic variants related to immune function and inflammation showed a better response to microbiota transfer therapy (MicTT) than those who did not have these variants [76]. Thus, genetic variations related to immune function and inflammation may influence GM, which, in turn, can have a bearing on the development of ASD [73]. Importantly, an interaction between GM and host genes, either directly or indirectly, via epigenetic (discussed later) mechanisms may occur [77,78]. Therefore, beyond genetic predisposing factors, elucidating GM composition, evolution, and function could not only enhance our understanding of contributory factors to a variety of diseases, including ASD but could also suggest novel interventions.

### 3.4. Gut Microbiota—Neurotransmitters

GM may also indirectly influence the functioning of CNS by encoding genes for specific enzymes that catalyze the conversion of some substrates into neurotransmitters or their precursors. Thus, GM produces and responds to the same neurotransmitters such as serotonin, dopamine, noradrenaline, acetylcholine, GABA, and glutamate that the brain uses to regulate a variety of behaviors, including mood and cognition [79,80]. Indeed, an imbalance between these neurotransmitters is believed to result in many neuropsychiatric and/or neurological diseases such as ASD, AD, PD, anxiety, and mood disorders [63,76]. These neurotransmitters are byproducts of the food breakdown and/or may be directly produced and secreted by GM. Many bacteria have been found to be able to produce large quantities of mammalian neurotransmitters. For example, Bacillus mycoides and Bacillus subtilis produce dopamine and noradrenaline, whereas some strains of *E. coli* (*E. coli* K12) produce dopamine, noradrenaline, and serotonin. Also, many strains of Lactobacillus species are known to produce GABA, ACh, and histamine [81], as well as tryptophan hydroxylase, the enzyme that catalyzes the rate-limiting step in the synthesis of serotonin. Further proof of associations between dysbiosis and changes in neurotransmitters and short-chain fatty acids was observed in a prenatal valproic acid model of ASD [82].

Consequently, the modulatory effect of the GM on CNS was exploited for the development of specific probiotics that can improve several CNS disorders, including stress disorders [83], mood and cognitive impairments [84,85], autoimmune diseases [86], as well as ASD [87].

### 3.5. Gut Microbiota—Short Chain Fatty Acids (SCFAs)

As briefly alluded to earlier, GM can interact with CNS via several pathways: First, via produced metabolites. Second, directly via neurotransmitters, and third, indirectly by influencing the synthesis and release of enteric hormones. In fact, GM produces and releases crucial neuro-active metabolites that play the role of neuromediators and neuromodulators. Among these, short-chain fatty acids (SCFAs), aromatic amino acids, and bile acids are the main substances affecting the nervous system. SCFAs are the main metabolites produced by the microbiota in the large intestine via the anaerobic fermentation of indigestible polysaccharides such as dietary fiber and resistant starch [88,89]. They have been associated with many different physiological processes, from GI functions to immune functions and CNS development and maturation [89,90]. Indeed, decreased levels of SCFAs have been associated with degenerative diseases such as PD [91], anorexia nervosa [92], and ASD [93]. Alterations of SCFAs (acetate, propionate, and butyrate) levels have also been reported in mood and anxiety disorders, which are commonly associated with ASD [94]. GM also has an important role in maintaining the integrity of the blood–brain barrier (BBB) [67,95,96]. BBB, a semipermeable endothelium, is a highly selective membrane preventing solutes and potentially harmful elements in the circulating blood from freely entering the CNS. SCFAs, however, can directly impact BBB integrity. This is evidenced by the impairment of the BBB in germ-free mice and its restoration by recolonization with *Clostridium tyrobutyricum*, which produces the SCFA (butyrate) and up-regulates the tight junction proteins [97].

### 3.6. Gut Microbiota—Microglia

As mentioned, inflammation has been linked to the pathogenesis of ASD. Microglia, which are the immune cells of the brain, play a critical role in the inflammatory response in CNS. One proposed mechanism for microglial activation leading to neuroinflammation in ASD involves the disruption of GBA. GM plays a critical role in regulating the immune system, as evidenced by the fact that dysbiosis, or alterations in the composition of GM, can lead to an inflammatory response in the gut, which can propagate to CNS via a variety of mechanisms, including the vagus nerve and circulating cytokines [98,99]. Indeed, it is now well recognized that microglia and astrocytes underlie neuroinflammation and synaptic susceptibility in ASD [100].

In addition to dysbiosis, other factors such as environmental toxins, including heavy metals, especially lead, which will be discussed in the following section, have also been implicated in microglial activation and neuroinflammation in ASD [101,102,103]. The activation of microglia and the subsequent release of pro-inflammatory cytokines can lead to synaptic dysfunction and neuronal damage, contributing to the cognitive and behavioral symptoms of ASD [42,43,44,104].

### 3.7. Gut Microbiota—ASD

**Figure 2:**
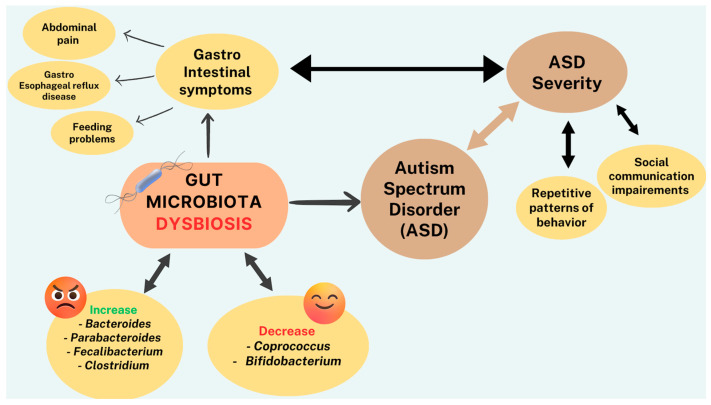
Schematic diagram depicting the influence of gut microbiota (GM) on ASD and GI symptoms associated with severity of the disorder.

Gastrointestinal symptoms have been vastly described in patients with various neurological diseases, including ASD [105]. Children and adolescents suffering from ASD report abdominal pain, constipation, gastroesophageal reflux, and feeding problems [106,107,108]. A large prospective cohort study showed differences in the stooling patterns and feeding behaviors as early as 6 months in children who were later diagnosed with ASD [108,109]. These GI comorbidities are more common and more often persistent in children with ASD than in children with other developmental delays [108,109]. Gastrointestinal symptoms seem to correlate with ASD severity [105,107,110]. An open-label study on Microbiota Transfer Therapy (MicroTT) supports the effectiveness of this therapy as significant improvement was noted, which lasted at least 8 weeks after the treatment [111]. Additionally, a recent systematic review and meta-analysis on microbiome components in ASD children showed multiple differences between the microbiota of neurotypical children compared to ASD patients [105]. For instance, increased levels of *Bacteroides*, *Parabacteroides*, *Faecalibacterium*, and *Clostridium* and decreased levels of *Coprococcus* and *Bifidobaterium* were detected. There was also an augmentation in the *Bacteroidetes*/*Firmicutes* ratio compared to controls, with an elevated *Bacteroidetes* and *Firmicutes* level overall. *Proteobacteria* and *Tenericutes* concentrations were also found to be higher in the microbiota of children suffering from ASD compared to neurotypical children [105,112]. In all, the levels of harmful microbiota were increased, whereas the levels of beneficial microbiota were decreased in patients with ASD (Figure 2). Interestingly, some species of *Bifidobacterium* produce GABA; hence, diminution of this bacterium in the microbiota may be a possible explanation for the low level of this inhibitory neurotransmitter in ASD patients. This is also consistent with the findings that neuromediators and/or neurotransmitters and their precursors synthesized in the gut may have a direct bearing on the levels of the same in the brain [67].

It is noteworthy that not only gut microbiota, but also the GI tract, play a critical role in the pathophysiology of ASD. This is because the intestinal barrier permeability components, from the mucosal layer to the junction systems, serve as the body’s first barrier against toxic metabolites. Thus, an impaired intestinal barrier increases inflammatory responses and positively correlates with ASD manifestations. In this context, an increase in markers of epithelial damage has been linked to an increase in ASD severity in children [113]. Moreover, since children with ASD have distinct microbiota composition, it has been suggested that this may be used as a biomarker or screening tool for ASD [105,114,115].

## 4. SCFAs-ASD

SCFAs such as butyrate, acetate, and propionate are metabolites produced by gut bacteria during the fermentation of dietary fibers and play a crucial role in the bidirectional communication between the gut and the brain [25,113,116,117]. These compounds can cross the blood–brain barrier and interact with neuronal and glial cells, modulating various functions, including neurotransmitter synthesis, energy metabolism, and inflammation. Indeed, SCFA alteration is believed to be directly involved in the development of ASD manifestations and comorbidities [117]. Moreover, neuronal signaling may be affected via activation of the vagus nerve, microglia, as well as intestinal T cells [116].

It is of relevance to note that propionic acid (PPA), a highly neurotoxic SCFA, is produced by Bacteroidetes [112,118,119]. PPA can cause alterations in neurotransmitter levels, including decreased levels of GABA and increased levels of glutamate in the brain [120]. Moreover, it can lead to gliosis, a neuro-inflammatory response [118]. Curiously, high levels of Bacteroidetes and elevated PPA levels were reported in children suffering from ASD [121,122]. Additionally, PPA administration used to generate a rodent model of ASD resulted in brain morphological changes, including alterations in the size and structure of the cortex, hippocampus, and amygdala, brain regions that are associated with the regulation of social behavior and cognition [123].

Beyond SCFAs’ involvement in ASD development, recent studies have shown an interesting correlation between ASD and various bacterial metabolites, such as para-cresyl sulfate (pCS) and 4-ethylphenyl sulfate (4EPS) [25]. However, the exact role of these metabolites in the pathophysiology of ASD has yet to be established [25].

## 5. Lead (Pb)

Pb is a naturally occurring heavy metal with numerous industrial applications, which has caused its ubiquity in the environment and exposed the population, particularly children, to it [124]. One of the most common sources of exposure for children in the US is the ingestion of Pb-contaminated dust and soil from deteriorating lead-based paint in houses constructed earlier than 1978. Pb is highly toxic as low levels of exposure can have harmful effects, especially on the brain. It enters the body primarily via inhalation and ingestion, although minimal amounts can penetrate the skin [125,126]. Pb exposure has been linked to a range of adverse health outcomes, including intellectual disability, behavioral and attentional problems, and developmental delays. Even acute exposure to Pb can cause encephalopathy and lead to coma, convulsions, and even death. Children who survive severe lead poisoning may be left with intellectual disabilities and behavioral disorders [127]. Indeed, it was reported that early (childhood) Pb exposure causes a reduction in brain volume in adulthood [128]. A study using functional MRI evaluated the impact of chronic Pb exposure during infancy and early childhood development and found impairments in cognitive functions such as memory, intelligence, language, decision-making capacity, and social skills, as well as decreased activation in language areas (left frontal cortex adjacent to Broca’s area and left middle temporal gyrus that include Wernicke’s area), whereas the right hemisphere homolog of Wernicke’s area was enhanced in these subjects, confirming that Pb exposure affects the brain function and organization [129]. Another study conducted by The Centers for Disease Control and Prevention (CDC) in the US in children aged 6 to 16 years found that even a low level of Pb exposure, as measured by blood Pb concentration, was associated with deficits in cognitive and academic skills, suggesting that there is no safe level of Pb exposure for children and that Pb exposure should be considered as a public health priority [130,131]. Similarly, prenatal Pb exposure is associated with deficits in executive function and areas of planning and decision-making mediated by the frontal lobe [132]. Finally, and importantly, Pb is suspected of contributing to the development of ASD [126,133].

### 5.1. Pb-ASD

Indeed, the causal association between Pb exposure and ASD is strongly supported by a large body of epidemiological data and is consistent with the toxicological profile of Pb, especially in children. For example, as early as 2013, a relationship between perinatal Pb exposures and ASD was reported [134]. In 2016, Dickerson and colleagues suggested not only an association between ambient Pb concentrations and ASD prevalence but also a possible synergistic effect of Pb with other metals, such as mercury and arsenic, in ASD [135]. More convincingly, a prospective study evaluating the relationship between low-level Pb exposure and autistic behavior in Korean school-age children concluded that “even low blood lead concentrations at 7–8 years of age are associated with more autistic behaviors at 11–12 years of age” [136]. Another compelling study in twins demonstrated the etiological relevance of early life exposure to Pb and ASD manifestation, notwithstanding the shared genetic risk factors of twins [137]. Further confirmations of the Pb-ASD link were provided by various investigators in different countries, demonstrating a strong correlation between Pb levels in hair and ASD symptoms [138,139,140,141,142]. Hence, a causal association between Pb exposure in children and ASD is supported by a preponderance of epidemiological studies [126,133].

### 5.2. Pb—Calcium—Neurotransmitters

Pb affects several neuronal pathways, including cholinergic, dopaminergic, GABAergic, and glutamatergic systems. One of the main targets of Pb with wide-range consequences is its disruption of calcium-dependent pathways. It is well known that calcium (Ca) is vital in numerous molecular pathways as the crucial second messenger, and without which, cellular response may not occur [143,144,145]. Pb can compete with Ca for binding sites on Ca-dependent channels and transporters and hence disrupt Ca homeostasis, which can lead to impairments in Ca-dependent neurotransmitter systems such as GABA and glutamate. Indeed, it has been hypothesized that lead-induced alterations in synaptic function and disruption of the delicate balance of excitatory and inhibitory neurotransmission during critical periods of brain development could contribute to the pathogenesis of ASD [146].

Additionally, disruption of molecules involved in synaptic transmission, neuronal survival, and plasticity, as well as interference with calcium-dependent enzymes and intracellular signaling involved in the regulation of gene expression, protein synthesis, and cell proliferation, can have profound effects on neuronal circuitries and hence lead to behavioral abnormalities [133]. Pb can also disrupt or damage the BBB, which can further exacerbate its neurotoxicity. Since the BBB of an immature (developing) brain is more vulnerable to damage [147], the effects of Pb may be even more pronounced in this population [133].

### 5.3. Pb—Neuroinflammation

One of the ways Pb may affect the brain is via its inflammatory effects via interaction with microglia, altering synaptic function and connectivity, causing changes in neurotransmitters and abnormal neural development. Microglia, considered the local CNS immune cells, play an important role during CNS development by shaping neuronal connectivity and supporting gliogenesis and myelination [103,148]. However, the overactivation of microglia can lead to the production of pro-inflammatory mediators, leading to neuroinflammation [149]. Indeed, a reactive state of glia referred to as “gliosis” is indicative of neuroinflammation and is considered the pathological hallmark of all types of neurodegenerative diseases [150]. Gliosis may be induced by a number of toxicants, including Pb [151]. Therefore, Pb, via microglia activation and induction of neuroinflammation, can contribute to ASD manifestation [151]. Other effects of Pb regarding mitochondrial damage (discussed below) may be additional mechanisms of how Pb exposure can lead to ASD.

### 5.4. Pb—Mitochondria

Mitochondria, the powerhouse of the cellular energy machinery and essential in life-sustaining processes, is one of the most sensitive organelles, damage to which can lead to serious consequences. In this regard, Pb-induced mitochondrial damage/dysfunction can result in pathological processes, including ASD [126]. Mitochondrial damage following Pb exposure can result in oxidative stress, apoptosis, and autophagy, as well as neuroinflammation, all of which may lead to neuropathology, including ASD [126,152]. In addition, the capability of Pb to disturb Ca homeostasis (discussed above) can contribute to its induction of ASD [152]. Specific pathways and mechanisms that lead to mitochondrial damage by Pb are extensively discussed in a recent review [152].

### 5.5. Pb—Microbiota—ASD

GM is an important, if not a critical, player in the maintenance of general health, including behavioral functions such as social interaction and cognition, impairments of which are hallmarks of ASD. Pb, in addition to the mechanisms contributing to ASD pathology, may also directly interact with GM and provide an alternate route or mechanism [153]. This relatively new discovery is a rather important advance in our understanding of ASD for two reasons. First, practically all the routes or mechanisms described so far for Pb may also be influenced by GM. Second, since GM manipulation appears to be much simpler compared to pharmacological or behavioral interventions that are currently used, new therapeutics for ASD may be suggested. Below, following some discussion of Pb-GM interaction, potential novel treatments are also touched upon.

GM, as any other organ in the body, carries distinct functions, some of which may provide protection against many toxicants, including heavy metals such as Pb. However, this system can also be overcome by excess exposure to a toxicant. Such disruption of GM or dysbiosis can lead to severe health consequences, including neuropsychiatric and/or neurodegenerative disease discussed above. For example, Pb exposure has been shown to alter the composition of GM in animals and humans, leading to changes in the abundance and composition of certain bacterial taxa and metabolic pathways. These changes may result in immune dysfunction and/or neurotoxicity. Sadykov and colleagues first discussed the dysbiosis induced by Pb toxicity, where they reported that oral Pb exposure for 2 weeks altered the Phyla and Genus of the bacteria in rats [154]. Similarly, exposure of zebrafish to Pb also resulted in significant changes in the microbial richness and diversity with a marked increase in Firmicutes ad Bacteroidetes (inflammatory markers) and simultaneous reduction in Proteobacteria and Fusobacteria (anti-inflammatory markers) [155]. A more recent study in zebrafish also found elevated levels of Pseudomonas, Halomonadaceae, Arcobacter, and Polaribacter (all considered detrimental bacteria) following exposure to Pb [156].

The further intricate relationship between Pb-GM-ASD is supported by the findings that there is a net increase in Desuffaoibrio, Turici bacter, and Ureaplasma, whose abundance was negatively correlated with cognitive functions, in Pb-exposed mice [157]. Moreover, a 2023 study established the central role of prenatal exposure to Pb in the alteration of the offspring’s gut microbiota and impairment of neurological functions, including neuro-developmental deficits such as learning disabilities and memory loss [158]. Thus, potential intervention in Pb-induced ASD-like symptoms may be achieved by manipulating GM prenatally or in the early postnatal period.

Exposure of Japanese quails for 49 days to Pb resulted in a remarkable increase in abundance of Bacteroides—associated with inflammation and metabolic disorders, and a reduction in *Faecalibacterium* and Bifidobacteria—considered to be beneficial gut bacteria. Pb exposure was also accompanied by disrupted intestinal structure and altered immune status [159]. These and other studies confirm the negative impact of Pb exposure on GM, where a reduction in beneficial bacteria such as *Faecalibacterium* and Bifidobacteria and an increase in potentially harmful bacteria like Bacteroides are observed [160].

**Figure 3:**
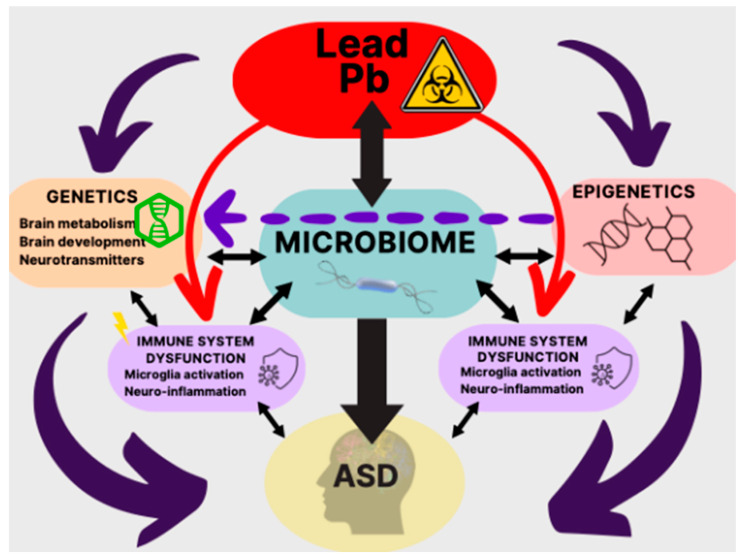
Schematic diagram depicting sequence of events initiated by lead (Pb) and leading to ASD. Specifically, Pb may affect the genetics, epigenetics, and gut microbiota, all of which, directly or indirectly, via immune system dysregulation, can lead to ASD.

Interestingly, Pb-induced dysbiosis may be time-dependent as dysbiosis was more prominent in the first 4 weeks of Pb exposure compared to the last 4 weeks, possibly due to the ability of GM to adapt and provide compensatory mechanisms to mitigate the negative effects of Pb [161]. As for the specific mechanism of Pb interaction with GM, it is likely that Pb causes a decrease in the gene encoding nitrite reductase (NADPH), a necessary enzyme for detoxification [160]. It is also noteworthy that patients with high levels of Pb in their urine had a lower abundance of Prevotella, a bacterium reportedly associated with anti-inflammatory properties [161,162,163]. Thus, an abundance of evidence from human or animal models confirms that Pb exposure can lead to dysbiosis, which can, in turn, lead to abnormal social or cognitive functions, hallmarks of ASD [164] (Figure 3).

It should also be noted that other divalent metals, as well as lanthanides, can alter microbiomes. However, the level of these divalent metals is extremely low, and so far, no incidence of physiologically relevant dysbiosis in relation to these elements and hence their involvement in ASD has been reported. Lanthanides, also called lanthanoids, consist of any of the series of 15 consecutive chemical elements in the periodic table, from lanthanum (atomic number 57) to lutetium (atomic number 71). These, together with scandium and yttrium, are referred to as “rare earth metals or elements.”

### 5.6. Pb—ASD—Therapeutics

Aside from manipulation of neurotransmitters and other culprits such as inflammation and oxidative stress implicated in ASD, several other treatment options may also be afforded by pre- or post-natal interventions via GM. It is of relevance to note that probiotics refer to live microorganisms that create a health benefit for the host, whereas prebiotics are components of food that are not necessarily digested by humans but essentially feed and promote beneficial bacteria in the gut [165]. Thus, dietary supplementation with a galactooligosaccharide (GOS), considered a prebiotic, was shown to promote fecal Pb excretion and reduce Pb accumulation in the blood and tissues of mice, suggesting that GOS can be considered a potentially protective prebiotic against Pb toxicity [166], and hence of therapeutic potential in ASD. Moreover, treatment of pregnant women, children, or adults with sodium butyrate or other probiotics, alone or in combination with other proposed effective agents such as antipurinergics, or peptidergic (e.g., oxytocin) or the amino acid taurine, may be suggested [46]. This suggestion is in line with established microbiome influences on neuro-immune interactions and recent advocacy of probiotics use as a treatment strategy for multiple sclerosis, another neurodegenerative disease [167,168].

Although a definite link between GM dysbiosis and ASD is well established, clinical trials based on microbiota therapeutics (probiotics, prebiotics, Fecal Microbiota Transplantation) and ASD improvement have yet to be confirmed [153]. In the same context, stem cell therapy has also been advocated [169]. In the latter case, a significant improvement in ASD scales (VABS: Vineland Adaptive Behavior Scales; CARS Childhood Autism Rating Scale) was not considered as full proof, but supportive evidence was noted [169]. Clearly, further controlled clinical trials are needed to support such interventions.

Additionally, as mentioned above, the use of vitamin B6 supplementation has also been recommended for ASD [59]. However, as our knowledge of the neurobiological substrates of this disorder expands, more novel interventions could be anticipated. In this regard and in lieu of the discussed mechanism, ameliorative effects of antioxidants, anti-inflammatory compounds, or chelators alone or in combination may emerge as a new perspective for therapeutic interventions against Pb-induced neurotoxicity [124]. In this regard, the protective effects of green tea supplementation against Pb-induced neurological toxicity in mice were recently reported. Significant improvements were noted in neurobehavioral responses and locomotory behaviors, as well as in biochemical indices [170]. In addition, the beneficial effects of a non-conventional method such as music therapy were recently reviewed [171]. It has been reported that a significant short-term improvement in various behaviors such as social interactions, social adaptations, and communications may be obtained by music therapy, hence making it a potential adjuvant therapy for ASD [172,173].

## 6. Conclusions

ASD, a devastating developmental disorder affecting cognition and social interaction, remains a significant medical challenge. The severity of the spectrum is mainly based on the level of social interaction, communication, and behavioral impairments such as restricted or repetitive behaviors. Although intellectual disability is not a diagnostic criterion; when present, however, it imparts an added complexity for the caregivers and the patient. Its genetic basis is highly heterogeneous, and to date, hundreds of genes have been identified. Thus, it may be either inherited or caused by novel mutations. Nonetheless, genetics may account for 10–20% of all ASD cases. Thus, a combination of genetic, epigenetics, immune system, gut microbiota (GM), and environmental exposure to toxic substances such as lead (Pb) are the main players in its etiology. Pb is a toxic heavy metal that has been linked to a wide range of negative health outcomes, including anemia, encephalopathy, gastroenteric diseases, and, more importantly, cognitive and behavioral problems. Recent studies have revealed that Pb exposure can disrupt GM, which is essential for maintaining overall health. Indeed, GM disruption, commonly referred to as dysbiosis, has been linked with numerous neurologic diseases, including ASD.

Neurobiological substrates include smaller cell size but increased density in the hippocampus, limbic system, entorhinal cortex, and amygdala, as well as neuroinflammation and mitochondrial damage. The current hypothesis suggests that ASD symptomatology is driven by four brain regions controlling social behavior. These include amygdala, orbitofrontal cortex (OFC), temporoparietal cortex (TPC), and insula. Regarding the neurotransmitter systems, Ca-dependent GABA and glutamate, as well as serotonin, dopamine, and acetylcholine, have been implicated. SCFAs, the main metabolites produced by the microbiota in the large intestine, have been associated with many different physiological processes, from GI functions to immune functions and CNS development and maturation. Hence their implication in ASD.

Thus, based on our current understanding of the disease process, its neurobiological substrates, as well as the involvement of GM, novel targets such as receptor stimulation (e.g., alpha7 nicotinic agonists) or GM manipulation via SCFAs (e.g., butyrate) and pre- and pro-biotics, as well as controlling Pb toxicity could be suggested. Further research in this area could lead to yet more novel pharmacotherapies for ASD.
